# FibROAD: a manually curated resource for multi-omics level evidence integration of fibrosis research

**DOI:** 10.1093/database/baac015

**Published:** 2022-03-09

**Authors:** Yu-Zhe Sun, Yong-Fei Hu, Yan Zhang, Shu-Yi Wei, Bei-Lei Yang, Ying-Ping Xu, Zhi-Li Rong, Dong Wang, Bin Yang

**Affiliations:** Dermatology Hospital, Southern Medical University, No. 2 Lujing Road, Yuexiu, Guangzhou 5100091, China; Dermatology Hospital, Southern Medical University, No. 2 Lujing Road, Yuexiu, Guangzhou 5100091, China; Dermatology Hospital, Southern Medical University, No. 2 Lujing Road, Yuexiu, Guangzhou 5100091, China; Dermatology Hospital, Southern Medical University, No. 2 Lujing Road, Yuexiu, Guangzhou 5100091, China; Dermatology Hospital, Southern Medical University, No. 2 Lujing Road, Yuexiu, Guangzhou 5100091, China; Dermatology Hospital, Southern Medical University, No. 2 Lujing Road, Yuexiu, Guangzhou 5100091, China; Cancer Research Institute, School of Basic Medical Sciences, Southern Medical University, No. 1023-1063 Southern shatai Road, Baiyum, Guangzhou 510515, China; State Key Laboratory of Organ Failure Research, National Clinical Research Center of Kidney Disease, Key Laboratory of Organ Failure Research (Ministry of Education), No. 1023-1063 Southern shatai Road, Baiyum, Guangzhou 510515, China; Dermatology Hospital, Southern Medical University, No. 2 Lujing Road, Yuexiu, Guangzhou 5100091, China; Department of Bioinformatics, School of Basic Medical Sciences, Southern Medical University, No. 1023-1063 Southern Shatai Road, Baiyum, Guangzhou 510515, China; Dermatology Hospital, Southern Medical University, No. 2 Lujing Road, Yuexiu, Guangzhou 5100091, China

## Abstract

Organ fibrosis represents a vital health threat that substantially contributes to yearly mortality rates. While a considerable amount of research has been conducted on fibrosis, these reports have only focused on specific organs as affected within distinct disorders. Accordingly, results from such studies have been unable to provide a comprehensive understanding of the pathological processes involved. Here, we describe the development of FibROAD, an open-access database that integrates evidence from fibrosis-associated disorders as obtained from both the literature and multi-omics data. This resource will greatly assist both researchers and clinicians in the comprehension and treatment of this condition. FibROAD currently involves an assembly of 232 strong evidence-based fibrosis-related genes (FRGs) as garnered from 909 PubMed publications and contains lists of multi-omics data from > 4000 samples including RNA-seq, single-cell RNA-seq, miRNA-seq, ChIP-seq, ATAC-seq MeDIP-seq and MBD-seq as obtained from 17 different organs in 5 species. Results from integrative analyses as obtained using FibROAD have demonstrated that FRGs can be indicators for a wide range of organ fibrosis and reveal potential pro-fibrotic candidate genes for fibrosis research. In conclusion, FibROAD serves as a convenient platform where researchers can acquire integrated evidence and a more comprehensive understanding of fibrosis-related disorders.

**Database URL**  https://www.fibroad.org

## Introduction

Fibrosis is a term used to describe the process of connective tissue deposition in response to organ injury and aging, and represents a condition that has attracted considerable attention and interest of late. As part of routine physiological processes, fibrosis can occur in almost all organs (1) but is particularly prevalent in the lung, liver, kidney, heart, bone marrow and skin. Moreover, fibrosis is associated with a number of essential biological processes, including aging (2) and wound healing (3). Ordinarily, fibrosis serves as a reparative and protective process to sustain normal functioning of organs, however pathological states of this process can induce excessive scarring resulting in organ malfunction and even failure. Complications due to fibrosis are currently the leading cause of death in the industrialized world (4) and represent a major challenge to global health. In addition, the cosmetic disfigurements of scars or keloids (5) resulting from fibrosis have generated a global industry of substantial economic consequence. Based on these issues, fibrosis has emerged as a condition of increasing importance and will require a pooling of all available resources to achieve a more comprehensive understanding for research and clinical treatments directed at this disorder.

With the advent and development of high-throughput omics techniques, it is now possible to obtain a more comprehensive evaluation regarding processes involved with fibrosis. As achieved with the use of the application of single-cell transcriptomics, Xie (6) described gene expression patterns of pulmonary fibrosis within a murine model, while Liu (7) illustrated the immune landscape of renal fibrosis, with both studies providing results at much higher resolutions than that obtained with more traditional techniques. Moreover, by combining multiple omics approaches, it is possible to decipher more precisely the processes and network changes that occur during organ fibrosis (8, 9). Although these results have greatly expanded our view of fibrosis, they represent findings that only focus on isolated organs within specific conditions. In this way, they limit the capacity of achieving a broader overview of this complicated process. Therefore, pooling of all available resources to achieve a more comprehensive understanding of fibrosis would seem a critical endeavor for a more complete understanding of these processes.

Currently, only a limited number of databases are available that can provide investigative information regarding fibrosis. These databases are concentrated on either unique types of diseases, such as the Cystic Fibrosis Database, Fibromine (10) and PulmonDB (11), or on specialized fields of research or techniques, including cell identification (IPF Cell Atlas), genetic mutations (Cystic Fibrosis Mutation Database) and pharmacology (comparative Toxicogenomics Database) (12). However, as fibrosis involves a complicated bio-pathological process, it is imperative that investigators consider and investigate this condition from a more comprehensive perspective with integrated evidence. Currently, no such platform effectively satisfying these requirements exists.

In an attempt to bridge this gap, we developed FibROAD (Fibrosis-Related Omnibus for Archives and Datasets, https://www.fibroad.org). This resource enables researchers to browse and analyze a wide range of multi-omics data associated with fibrosis, based on a manually curated, evidence-based fibrosis-related gene set. The current version of FibROAD consists of a set of 232 Fibrosis-Related Genes (FRGs) carefully filtered as based on experimental evidence from PubMed publications and lists the omics data from researches on 4351 samples involving RNA-seq, single-cell RNA-seq, miRNA-seq, ChIP-seq, ATAC-seq MeDIP-seq and MBD-seq. With FibROAD, researchers are provided with a ‘roadmap’ enabling them to evaluate fibrosis from a more global perspective. We believe this database would be extensively used and significantly promote new avenues for future research in fibrosis.

## Results

### Contents and statistics of FibROAD

**Figure 1.**
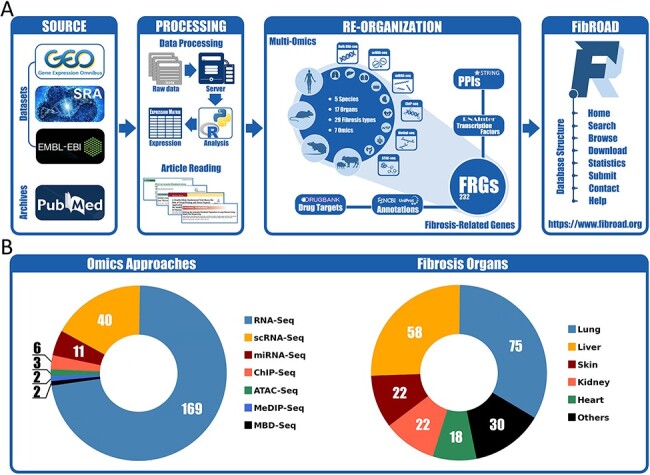
Workflow and statistics for the FibROAD database. (A) pipeline diagram depicting construction of the principal workflow of FibROAD. (B) Pie diagrams for the proportions of omic approaches (left) and organs (right) collected in FibROAD, with numbers indicating totals of projects.

The overall workflow for the construction of FibROAD is illustrated in Figure 1A. The major contents of FibROAD are divided into two parts: (i) fibrosis-related genes (FRGs), which consists of a gene list containing FRGs curated from PubMed publications and (ii) multi-omics evidence, which includes fibrosis-related high-throughput data collected from GEO, SRA and EBI-ENA databases.

With the use of these methods and criteria previously described, the current version of FibROAD incorporates 232 FRGs from 909 PubMed publications with strong experimental evidence and lists the experimental methods employed, functions (pro- or anti-fibrotic) and their literature sources (PubMed IDs). For gene descriptions, FibROAD introduces each FRG with its basic information and aliases collected from both NCBI-Gene and UNIPROT databases. In addition, FibROAD also provides information on FRGs related protein–protein interactions, transcription factor regulations and drug–target interactions, as obtained from STRING, RNAInter (13) and DrugBank databases, respectively.

For the Omics component (Figure 1B), FibROAD lists the data from 3599 RNA-seq (169 projects), 392 single-cell RNA-seq (40 projects), 252 miRNA-seq (11 projects), 64 ChIP-seq (6 projects), 18 MeDIP-seq (2 projects), 16 ATAC-seq (3 projects) and 10 MBD-seq (2 projects) samples as obtained from 227 independent projects in 5 different species (*Homo sapiens, Mus musculus, Rattus norvegicus, Sus scrofa, Acomys cahirinus*). Among these projects a total of 17 different sources of fibrotic organs are included, with lung (75), liver (58), skin (22), kidney (22) and heart (18) being the top five, and others (blood, muscle, intestine, blood vessel, bone marrow, eye, pancreas, oral cavity, gall bladder, spleen, respiratory tract and tendon) present in smaller numbers. Detailed dataset statistics are shown in Table 1. Notably, two projects involving COVID-19-associated fibrosis (lung fibrosis) were also included owing to the significance of the worldwide SARS-COV-2 outbreak.

**Table 1.** Dataset statistics of FibROAD

Organ	Species (count)	Fibrosis type (count)	Omics (count)	Organ	Species (count)	Fibrosis type (count)	Omics (count)
Lung	Homo sapiens (48)	IPF (25)	RNA-Seq (55)	Muscle	Homo sapiens (1)	FMD (1)	RNA-Seq (3)
	Mus musculus (27)	cystic fibrosis (8)	scRNA-Seq (19)		Mus musculus (3)	models (3)	scRNA-Seq (1)
	Rattus norvegicus (2)	ILD (3)	miRNA-Seq (2)				
	Sus scrofa (1)	other lung fibrosis (11)	ChIP-Seq (1)				
		models (37)	ATAC-Seq (1)				
Liver	Homo sapiens (15)	liver fibrosis (7)	RNA-Seq (45)	Eye	Mus musculus (1)	models (1)	RNA-Seq (1)
	Mus musculus (42)	models (54)	scRNA-Seq (8)				
	Rattus norvegicus (4)		miRNA-Seq (4)				
			ChIP-Seq (4)				
Kidney	Homo sapiens (1)	models (21)	RNA-Seq (16)	Gall bladder	Sus scrofa (1)	models (1)	RNA-Seq (1)
	Mus musculus (19)		scRNA-Seq (1)				
	Rattus norvegicus (1)		miRNA-Seq (2)				
			ChIP-Seq (1)				
			Methylation-Seq (1)				
Skin	Homo sapiens (14)	keloid (7)	RNA-Seq (14)	Oral cavity	Homo sapiens (1)	OSF (1)	RNA-Seq (1)
	Mus musculus (8)	hypertrophic scar (2)	scRNA-Seq (8)				
	Rattus norvegicus (1)	scleroderma (2)	miRNA-Seq (2)				
	Acomys cahirinus (1)	wound healing (5)	ATAC-Seq (1)				
		models (11)	Methylation-Seq (1)				
Heart	Homo sapiens (4)	cardiac fibrosis (1)	RNA-Seq (17)	Pancreas	Mus musculus (1)	models (1)	RNA-Seq (1)
	Mus musculus (12)	models (18)	scRNA-Seq (1)				
	Rattus norvegicus (1)		ATAC-Seq (1)				
	Sus scrofa (2)						
Blood	Homo sapiens (9)	cystic fibrosis (5)	RNA-Seq (6)	Respiratory tract	Homo sapiens (1)	cystic fibrosis (1)	RNA-Seq (1)
		liver fibrosis (2)	miRNA-Seq (2)				
		systemic sclerosis (1)	Methylation-Seq (1)				
		models (1)					
Intestine	Homo sapiens (3)	cystic fibrosis (1)	RNA-Seq (4)	Spleen	Mus musculus (1)	models (1)	RNA-Seq (1)
	Mus musculus (1)	intestinal fibrosis (2)	Methylation-Seq (1)				
	Rattus norvegicus (1)	models (2)					
Bone marrow	Homo sapiens (1)	myelofibrosis (1)	RNA-Seq (1)	Tendon	Homo sapiens (1)	tendon fibrosis (1)	RNA-Seq (1)
	Mus musculus (2)	models (2)	scRNA-Seq (2)				
Blood vessel	Mus musculus (2)	models (2)	RNA-Seq (2)				

Abbreviations: IPF—idiopathic pulmonary fibrosis; ILD—interstitial lung disease; FMD—facioscapulohumeral muscular dystrophy; OSF—oral submucous fibrosis

### Website interface of FibROAD

**Figure 2.**
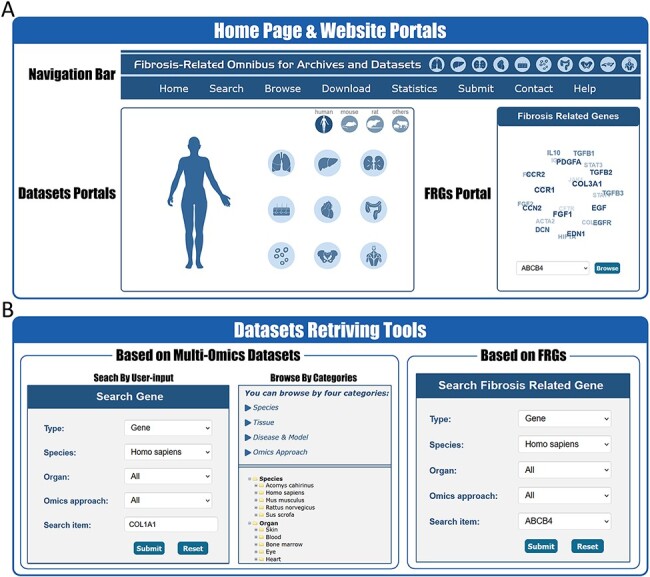
Website interface for FibROAD portals and dataset retrieving tools. (A) The home page of the FibROAD website was equipped with a navigation bar at the top and portals to enter dataset browsers within specific categories (left), as well as portals to enter for the page containing fibrosis-related genes (right). (B) The FibROAD dataset retrieving tools (Search and Browse), which can be accessed through the navigation bar on the home page. Users can retrieve relative information with either self-defined keywords (left) or with pre-set FRGs (right).

**Figure 3.**
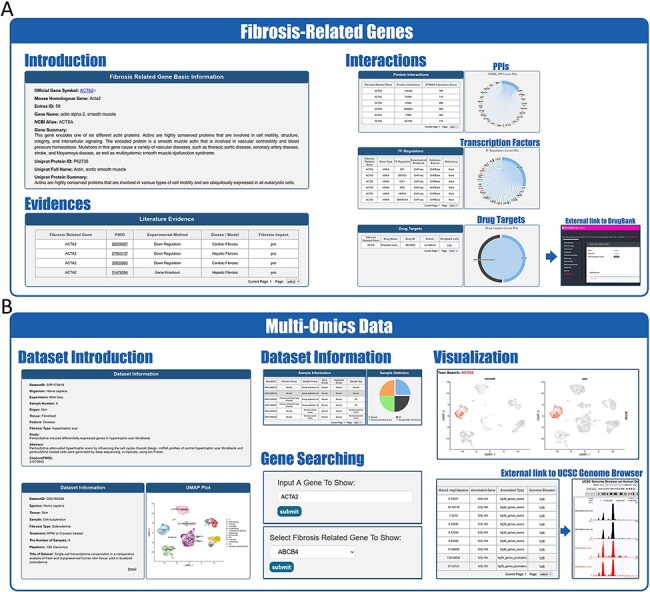
Website interface for FibROAD results and visualizations. (A) Result page for Fibrosis-Related Genes: gene introduction collected from both NCBI-Gene and UniProt databases are displayed in the basic information column, followed by literature evidence for the gene (left). Protein-protein interactions (right upper), transcription factor regulators (right middle) and drug targets (right lower) with the FRG are collected from String, RNAinter and DrugBank databases, respectively. An external link to the DrugBank database record for each target drug is provided. (B) Result page for omic data: retrieved datasets of types (RNA-seq, miRNA-seq, scRNA-seq, ATAC-seq, ChIP-seq, Methyl-seq) are shown in a similar pattern, with dataset introduction at the top (left panel), followed by a detailed sample information table (middle upper panel). A search tool for a self-input gene or FRGs within the dataset is provided (middle lower panel) and, once an item is submitted, search results and visualizations will be displayed below (right panel). For ATAC-seq/ChIP-seq/Methyl-seq, an external link to the UCSC Genome Browser is provided for genome tracks observation.

In order to better facilitate the retrieval of relative information in FibROAD, we constructed an open-access website that offers an opportunity for users to search, browse and download the data. On the home page (Figure 2A), the users are provided with portal links to either omics datasets or FRGs. By selecting icons of desired dataset categories on the dataset portal regions, users will be redirected to the Browse tool where results from filtered datasets are displayed. Within the FRGs’ portal region, all 232 FRGs are listed in the form of a combo-box, where users can select any FRG of interest and click the ‘Browse’ button to obtain basic information, protein–protein interactions, transcription factor regulations and drug targets for that specific FRG (Figure 3A).

FibROAD also offers a user-friendly portal to search gene expression profiles within the entire database to enable a more individualized use of the database (Figure 2B). Users can choose to either select a gene of particular interest or one from the FRGs list for a customized search (Search tool), with responses supplying statistical information on the expression of that gene. Internal links to specific datasets are provided along with these search results, allowing users access to more details regarding the expression of that gene. Moreover, FibROAD provides a file-directory-structured interface (Browse tool) for users to locate datasets in specific categories, including species, organs, experiments and diseases/models. Once a specific dataset is selected, users will be redirected to a page where detailed dataset information such as introduction, sample categories, distinct expression values (transcriptomic expression values or epigenomic peak concentration values) and data visualizations are displayed (Figure 3B). Moreover, on any result page, a convenient portal is available for users to input their desired gene or select any gene from the FRGs to review its expression and cell location (for scRNA-seq) within the dataset.

Other important functional components of FibROAD such as Download, Submit and the Help page can be accessed through the navigation bar of the website (Figure 2A).

### Fibrosis-related genes (FRGs) in FibROAD

**Figure 4.**
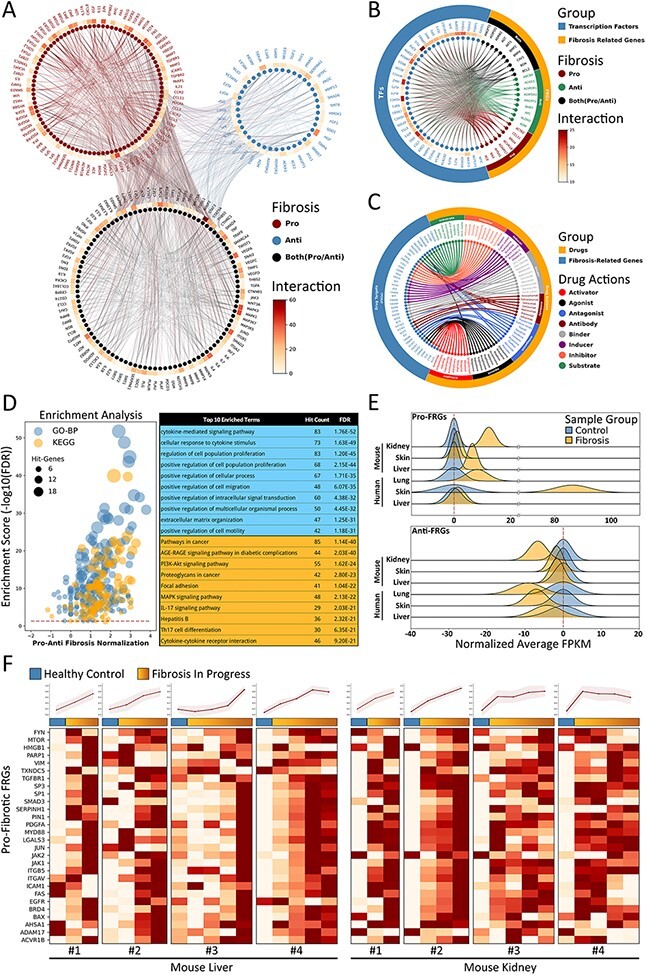
Fibrosis-Related Genes in FibROAD. (A) Circos plot for protein-protein interactions of all 232 FRGs as obtained from the String database. FRGs are divided into three groups (pro/anti/both shown in different colors) according to evidence contained within the literature. Interaction counts (edges number) for each FRG are presented with a circular heatmap around each circle. (B) Circos plot for several example transcription factors-FRGs regulations according to hTFtarget and RNAinter databases. Transcription factors are shown in orange whereas FRGs are divided into the three groups as described above and shown in steel blue. Regulation counts (edges number) for each transcription factor are shown with a surrounding circular heatmap. (C) Circos plot for several examples of drug targets according to the DrugBank database. Drugs are shown in orange whereas gene targets (FRGs) in steel blue. The eight different drug actions are presented using different colors. (D) Gene ontology (biological processes, steel blue) and KEGG (orange) enrichment analysis for FRGs (left panel). The pro/anti-fibrosis normalization (*x*-axis) was performed by calculating the *z*-score for pro- and anti-fibrosis FRGs within each term. The enrichment score (*y*-axis) was calculated with FDR from the hyper-geometry test. Red dashed line refers to the FDR threshold (0.05). The top 10 enriched terms of GO-BP (steel blue) and KEGG (orange) are shown in the right panel. (E) The average expressions (FPKM) of pro- (upper panel) and anti- (lower panel) FRGs in several datasets, with expressions of fibrotic groups (orange) normalized to that of control groups (steel blue). (F) Expressions (FPKM) of detected pro-fibrotic FRGs in datasets designed for fibrosis progression research. Results from healthy control groups are indicated in steel blue whereas fibrotic groups are displayed in orange gradients. Results of 4 independent datasets for mouse liver (left panel) and mouse kidney (right panel) are each illustrated in heatmaps, with normalized average expressions depicted in line plots at the top.

In an attempt to interpret the molecular essence of fibrosis, we provide a manually curated collection of 232 FRGs with inclusion criteria as described above, along with associated experimental evidence as resulting from 909 representative PubMed publications. According to the experimental evidence, FRGs can be divided into three functional groups, namely pro-fibrotic (91 genes), anti-fibrotic (41 genes) and both (100 genes, with either pro- or anti-fibrotic function as reported in different studies) (Figure 4A). It should be noted that these three categories were determined as based on current evidence from the literatures as filtered by our inclusion criteria, and would be updated if new evidence were to be reported in the future. By applying the PPI-relations from the STRING database with interaction scores >900, we found that FRGs were closely interactive with each other, showing an average of 13 linkages per gene. Moreover, we also found that many FRGs share common up-stream transcription factor regulators according to results from the hTFtarget (14) and RNAinter databases. Such findings suggests that a portion of FRGs may respond synergistically to similar cellular stimuli and functions (some examples of TFs-FRGs regulations are illustrated in Figure 4B). In addition, by integrating information obtained from the DrugBank database, we observed that many FRGs also serve as targets for therapeutic or experimental drugs with varied actions, such as agonist, antagonist, activator, inhibitor and binder (several examples of drug–FRGs interactions are illustrated in Figure 4C).

To further describe functions of these FRGs, we next performed an enrichment analysis for both Gene Ontology Biological Process (GO-BP) and KEGG Pathway with use of the entire list of FRGs (Figure 4D). The top 10 enriched GO-BP and KEGG (FDR threshold set to 0.05) terms were highly concentrated in fibrosis-related processes regarding cytokines (15), cell migration (16) and extracellular matrix organization (17), as well as fibrosis-related pathways involving cancer (18) and focal adhesion (19). These results are in accord with findings from FRGs protein-protein interactions and TF regulations, demonstrating that FRGs provided by FibROAD comprise a closely correlated gene set with regard to fibrosis-associated processes and functions.

Next, we assessed the average expressions (in terms of FPKM) of FRGs in several RNA-seq datasets collected in FibROAD to determine if the FRGs list proposed would effectively describe the features of fibrosis (Figure 4E and F). When summarizing results from human lung, skin and liver fibrosis, as well as kidney, skin and liver fibrosis of samples from mice, we found that pro-fibrotic FRGs (upper panel of Figure 4E) were expressed in higher levels within fibrotic samples as compared to that obtained from samples of healthy controls, while average anti-fibrotic FRGs expressions (lower panel of Figure 4E) are lower when compared than that obtained in control groups. In order to determine whether FRGs could provide a dynamic description of procedures involved with fibrosis, we evaluated the expressions of intersected pro-fibrotic FRGs in eight datasets (four liver and four kidney datasets) whose projects were designed to show the dynamic transcriptomic changes that occur during murine fibrosis model induction. As revealed from the heatmap results presented in Figure 4F, the pro-fibrotic FRGs detected in all eight datasets show an increasing expression pattern as the induction of fibrosis progresses (except for the last dataset whose expression remained stable after induction). The normalized average expressions of all genes in each sample are illustrated in the line plots above the heatmaps. These results demonstrate that the FRGs as proposed in FibROAD can be used as indicators for different organ fibrosis at the trancriptomic level.

### Application of FibROAD to identify potential target genes for organ fibrosis

**Figure 5.**
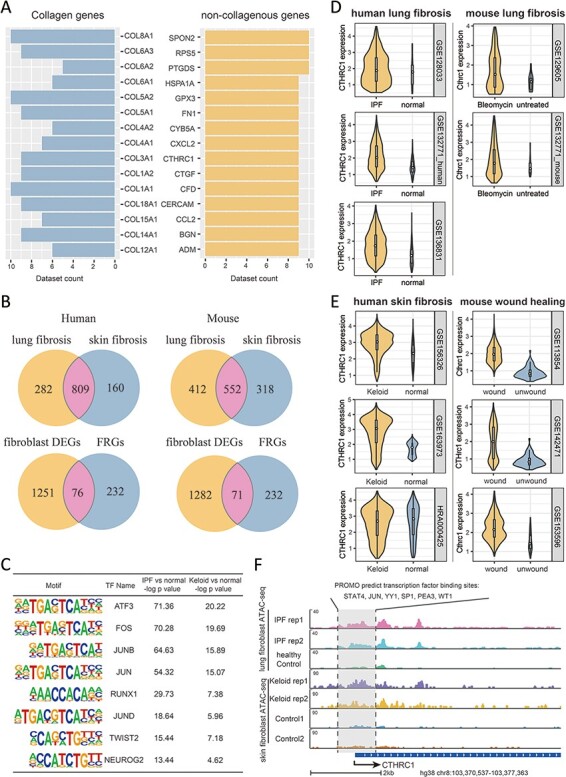
Multi-omics explorations with FibROAD indicate that CTHRC1 may be a potential candidate gene for skin fibrosis. (A) Bar graphs showing the top 15 most extensively and differentially expressed collagenous and non-collagenous fibroblast genes in scRNAseq datasets as collected with FibROAD (filtered by Wilcoxon Rank Sum test with log fold-change cutoff of 0.5 and *P* < 0.05). (B) Venn plots showing intersections of differentially expressed genes (DEGs) between lung and skin fibroblasts, in humans and mouse respectively (upper 2 Venn plots), and the intersections between DEGs and FRGs in the two species (lower 2 Venn plots). (C) Transcription factor motif enrichment analysis of differential chromatin accessibility regions located in the promotor region of the 55 commonly differentially expressed FRGs with ATAC-seq fibroblast datasets. (D-E) Violin plots showing significant differences in CTHRC1 expressions within lung (D) and skin (E) fibroblasts between the fibrotic group and healthy controls, in humans (left panel) and mice (right panel). (F) Representative genome browser tracks comparing CTHRC1 ATAC-seq signals in fibroblasts between the fibrotic group and healthy controls.

To go further into the molecular features of fibrosis and their associations with FRGs, we integrated a series of multi-omics data as collected in FibROAD from different organ sources, including lung, liver, kidney, heart and skin. As fibroblast is the key cellular component associated with fibrotic processes (20), we first examined transcriptomic alterations of fibroblast with use of all scRNA-seq datasets in FibROAD. By setting the logFoldchange (fibrosis groups against normal controls) and *P*-value thresholds to 0.5 and 0.05, respectively, a considerable number of genes were found to be differentially expressed (DEGs) in multiple datasets, with the top 15 extensively expressed collagenous and non-collagenous genes illustrated in Figure 5A. Within these results a high degree of DEGs overlap was particularly notable within fibroblasts of lung and skin (upper two venn diagrams in Figure 5B), which also share 76 and 71 of genes with FRGs in human and mice, respectively (lower two venn diagrams in Figure 5B), and the intersection of these two groups further reveals 55 differentially expressed FRGs in common. To decipher the potential regulators associated with these differentially expressed FRGs, we performed a transcription factor motif enrichment analysis with use of the ATAC-seq fibroblast datasets for lung fibrosis (idiopathic pulmonary fibrosis) and skin fibrosis (keloid) in FibROAD. The results of this analysis provide a prediction for several potential TF motifs on the promotor region of these differentially expressed FRGs (Figure 5C), among which the AP-1 family (especially the JUN family) were found to be significantly enriched. Interestingly, as a pro-fibrotic FRG, JUN was found among the conserved FRGs which could describe the dynamic alterations during fibrosis progress (Figure 4F). Moreover, use of the TFs-FRGs regulation network tool embedded in FibROAD revealed that the JUN family regulates collagen triple helix repeat containing 1 (CTHRC1), one of the top 15 extensively expressed non-collagenous genes (Figure 5A). And, findings from recent reports have indicated that CTHRC1 influences a special pro-fibrotic signature in both lung (21) and heart (22) fibroblasts, which could also be substantiated by correspondent scRNA-seq datasets in FibROAD (Figure 5D). When searching for the expression profile of CTHRC1 in other tissues with fibrosis, we found similar effects within the skin, with CTHRC1 being elevated in both keloid and wound healing fibroblasts (Figure 5E). To further assess the means for this regulation of CTHRC1 expression in the skin, we examined chromosomal accessibilities of the CTHRC1 promoter region with use of ATAC-seq datasets involving keloids and lungs in FibROAD (Figure 5F). The results showed that in both fibrotic groups the CTHRC1 promoter region was more accessible as compared with that observed in healthy controls. As expected, JUN was present among the 6 TF binding site predictions (STAT4, JUN, YY1, SP1, PEA3 and WT1) of PROMO. In conclusion, after a combining integrated multi-omics analysis with FibROAD, we found that JUN/CTHRC1 expression and interaction might represent a potential molecular mechanism involved with the promotion of multiple organ fibrosis, especially in lung and skin. Accordingly, increased attention should be directed toward the relationship of these genes with fibrosis in future research.

## Discussion

Fibrosis, a condition resulting from extracellular matrix accumulation in response to organ injury, represents a major causative factor for a number of diseases, such as idiopathic pulmonary fibrosis (23), cystic fibrosis (24) of the lung, hepatic cirrhosis (25) of the liver, post-infarction cardiac failure (26) of the heart and keloid (5) of the skin. Therefore, this condition has attracted considerable attention of late, with >340 000 fibrosis-related articles being published since 1872, and over 50% of these in the past decade, according to PubMed. We made a systemic review on the currently existed online resources regarding fibrosis (Supplementary File 1), which found that, however, no databases are capable of providing fibrosis-related information within both the literature and multi-omics levels across multiple organ origins. Given this lack of information along with the significance of this condition and potential for future research in the area of fibrosis we created FibROAD.

The aim of our database is to facilitate researchers in the field of fibrosis by providing an integration of high-quality omics data of the field, as well as curating a highly confidential gene set to characterize the essential process of organ fibrosis. We provided a convenient platform and tool for investigators to relate evidences from different aspects of fibrosis researches. With the current version of FibROAD, users could explore fibrotic information such as gene expressions, epigenomic regulations, protein interactions and drug targets from 232 FRGs as garnered from PubMed publications, and datasets for a total number of 4351 samples from multi-omics studies as obtained from 17 different organs in 5 different species, and hopefully reveal important regulation network and hub features underlying biological and pathological mechanism of fibrosis.

Although there have been some attempts to characterize the processes of fibrosis as achieved by summarizing a set of specific gene entities, either with use of text mining (27) or from aspects of a specific disease (11, 28), these studies have failed to do so as grounded on solid evidence-based data or from a broader range of organ characteristics. As one approach to rectify these deficits, we curated 232 FRGs from PubMed literature with strong experimental evidence into the current version of FibROAD, and documented specific gene functions, tissue origins and experimental techniques for each of these studies. With this approach, we identified genes with highly unified pro- or anti-fibrotic functions, and the presence of these genes was also substantiated with the multiple datasets as collected in FibROAD. Moreover, additional genes that can be differentially activated within different organs or environment scenarios were observed, suggesting that fibrosis is a complicated process which will require investigations at several levels. The integrated multi-omics data within FibROAD can provide one such mechanism for these investigations. Data comparison with similar web-based resources (Fibromine, IPF Cell Atlas, PulmonDB and TiRe) shows that the current version of FibROAD provides more fibrosis information regarding organs, species, omics approaches and FRGs references (Table 2). With this resource, investigators are provided with a convenient platform to browse and relate fibrosis-related information within categories of interest and, not only explore transcriptomic changes within a given range of fibrosis, but also their epigenetic regulation, topological relationships and medical interactions. In this way, this more integrated data source will lead to a more comprehensive understanding of the processes involved with fibrosis.

**Table 2.** Data comparison of FibROAD with similar web-based resources

		FibROAD	Fibromine	IPF Cell Atlas	PulmonDB	TiRe
Organs	Total count	17	1	1	2	2
	Type	lung, liver, skin, kidney, heart, blood, muscle, intestine, blood vessel, bone marrow, eye, pancreas, oral cavity, gall bladder, spleen, respiratory tract, tendon	lung	lung	lung, blood	lung, skin
Datasets	Total counts	233	110	6	79	N.A.
	Omics types	7	4	1	2	N.A.
	RNA-seq	169	15	0	4	N.A.
	scRNA-seq	40	16	6	0	N.A.
	miRNA-seq	11	0	0	0	N.A.
	ChIP-seq	6	0	0	0	N.A.
	ATAC-seq	3	0	0	0	N.A.
	MeDIP-seq	2	0	0	0	N.A.
	MBD-seq	2	0	0	0	N.A.
	Array	0	70	0	75	N.A.
	Proteomics	0	9	0	0	N.A.
Species	Total count	5	2	1	1	4
	Type	Homo sapiens, Mus musculus, Rattus norvegicus, Sus scrofa, Acomys cahirinus	Homo sapiens, Mus musculus	Homo sapiens	Homo sapiens	Homo sapiens, Mus musculus, Rattus norvegicus, Sus scrofa
FRGs	Gene counts (N.R.)	232	N.A.	N.A.	N.A.	498
	Reference counts (N.R.)	909	N.A.	N.A.	N.A.	607
	Organs (Ref.)	lung (172), liver (217), skin (37), kidney (156), heart (187), others^a^ (159)	N.A.	N.A.	N.A.	lung (196), skin (411)

Abbreviations: N.A.—not available; N.R.—non-redundant; Ref.—reference counts; FRGs—fibrosis-related genes; ^a^—details can be found online at FibROAD.

With the advent of new research directed toward fibrosis, in particular with that employing new techniques such as mass-spectrometry and post-transcriptional modification assays, substantial increases in valuable information regarding fibrosis will be revealed. Therefore, our FibROAD program will not only be critical in integrating this information but also will be continuously managed and upgraded to provide a long-term and current database of this information. As a result, we believe this database will be extensively used and significantly promote new avenues for future research in the area of fibrosis.

## Materials and methods

### Fibrosis-related genes curation and criteria

FRGs were collected from PubMed literature published prior to July, 2021, as retrieved using the terms ‘fibrosis’ or ‘fibrotic’ in the searches of Titles/Abstracts. The search results were initially filtered by limiting the literature type to ‘experimental research’ and ‘review with experimental references’. Then, evidence strength of the experiments in the literature was further evaluated, to isolate studies with strong evidence (gene knock-out/knock-in, gene overexpression, RNA interference, direct treatment with gene-coded protein, specific inhibition/activation, direct gene upregulation/downregulation). Any reported genes that satisfied the above criteria in at least two independent studies were considered as fibrosis-related genes and thus curated into the Fibrosis-Related Genes (FRGs) component of FibROAD. Together with the gene symbols, their representative references (2–8 studies) along with pro-/anti-fibrotic function in each reference and level of evidence were also collected.

### Omic data collection and criteria

High-throughput omic data were collected from GEO, SRA and EBI-ENA databases, published prior to February 2021 as achieved by searching the keywords ‘fibrosis’ or ‘fibrotic’. A manual screening of the retrieved results was performed to ascertain whether the subject and experimental design focused on fibrosis. Among the filtered omic approaches, RNA-seq, single-cell RNA-seq, miRNA-seq, ATAC-seq, ChIP-seq and Methylation-seq (MBD-seq and MeDIP-seq) were curated for further data processing.

### Omic data processing

#### RNA-seq

We used Hisat2 (v2.0.52) to build the index of the reference genome for different organisms and align the paired-end clean reads with the reference genome (29). Then, StringTie (v2.23) was used to count the read numbers mapped to each gene (30). Fragments Per Kilobase per Million (FPKM) of each gene were calculated based on gene length and read counts mapped to this gene. Differential expression was defined by a Benjamini–Hochberg-adjusted *P*-value (*q*-value/FDR) of <0.05 and fold change of >2 or <0.5.

#### Single-cell RNA-seq

Sequencing reads were examined by quality metrics, with transcripts mapped to a reference human (hg38) or mouse (mm10) genome and assigned to individual cells of origin according to cell-specific barcodes, using the Cell Ranger pipeline (10X Genomics). To ensure that PCR amplified transcripts were counted only once, only single UMIs were counted for gene expression levels (31). In this way, cell-gene UMI counting matrices were generated for downstream analyses. From each sample, unwanted variations and low-quality cells were filtered by removing cells with high and low UMI-counts (>6000 and <200). Gene expression levels for each cell were normalized by the total expression, multiplied by a scale factor (10 000) and log-transformed. Batches were then regressed out, and scaled *Z*-scored residuals of the model were used as normalized expression values. We defined the top 2000 most variable genes as based on their average expression and dispersion as being highly variable genes (HVG). Dimensionality of the data was reduced by performing a principal component analysis (PCA) on the HVG. To identify cell subpopulations, clustering was performed on PCA scores using significant PCs assigned by a randomization approach as proposed by Chung and Storey (32, 33). To cluster cells, a K-nearest neighbor (KNN) graph constructed on a Euclidean distance matrix in PCA space was calculated and then converted to a shared nearest neighbor (SNN) graph, in order to locate highly interconnected communities of cells (34). Cells were then clustered using the Louvain method to maximize modularity (35). To display data, the Unsupervised Uniform Manifold Approximation and Projection (UMAP) method was applied to cell loadings of selected PCs and the cluster assignments from the graph-based clustering were used. For cluster numbers >2, cluster-specific marker genes were identified by running the ‘find_all_markers’ Seurat function with parameters consisting of a logfc.threshold = 0.5 and test.use = ‘wilcox’. To identify differentially expressed genes between two clusters, we used the ‘find.markers’ Seurat function with a logfc.threshold = 0.5 and test.use = ‘wilcox’. All analyses described in this section were performed using Seurat R package version 3.0.1 (36).

#### ATAC-Seq/ChIP-Seq/MBD-Seq/MeDIP-Seq

After removing adaptors using Trimmomatic (37), reads were mapped to the reference human (hg38) or mouse (mm10) genome using Bowtie2 (38). Mapped reads of SAM output were converted to a BAM format and sorted by Samtools (39). Duplicate reads were removed using the default parameters of the Picard tools MarkDuplicates program (http://broadinstitute.github.io/picard/). Accessible regions and peaks of each sample were identified using MACS2 (40), with the narrowPeak files from the MACS2 output used for further analysis. The annotatePeaks.pl tool of the HOMER program (41) was used with the default parameters to annotate the location of identified peaks overlapping with genomic features. To identify regions with differential peak values, we used Diffbind with the processed alignment bam file from Bowtie2 and the narrowPeak file from MACS2 for each sample (42). For the MeDIP-Seq, differential coverage between experimental groups was calculated using the MEDIPS R package (43). The *P*-value from edgeR was used to determine significance of the difference between the two groups for each 100-bp genomic window (44). Windows with an edgeR *P-*values less than a specified threshold (*P* < 10–7) were considered as the initial start of the differentially methylated regions.

#### miRNA-Seq

Reads with low-quality or with a length <15 or >41 nt in the raw data were filtered to obtain clean reads. Clean reads were initially used for length distribution analysis in the reference genome. These clean reads were then aligned, subjected to BLAST database analysis (45), and searched against Rfam (v.10.1) (http://www.sanger.ac.uk/software/Rfam) (46) and GenBank databases (http://www.ncbi.nlm.nih.gov/genbank/). The noncoding RNAs annotated as tRNAs, rRNAs, small nuclear RNAs (snRNAs) and small nucleolar RNAs (snoRNAs) were excluded from the analysis. Known miRNAs were identified by alignment against the miRBase (v.22.1) database (http://www.mirbase.org/) with Bowtie (v1.1.1), and their expression levels were quantified with miRDeep2 (47). Differentially expressed miRNAs were identified as those meeting a threshold *P*-value <0.05 and a fold change >2.0. The fold change and *P*-values were calculated with the DEG algorithm (R, DESeq package) (48).

### Statistics and visualizations

All statistical calculations were performed with use of R (version 3.6.3) or Python (version 3.8.8) programs. Statistical significance was considered when adjusted *P*-values (*q*-value or FDR) or Wilcoxon Rank Sum test *P*-values were <0.05. Figures were plotted with use of either the ggplot2 library of R (version 3.6.3) or matplotlib package of Python (version 3.8.8) programs.

## Supplementary Material

baac015_Supp
