# Supplementary material for: FibROAD: a manually curated resource for multi-omics level evidence integration of fibrosis research
Source: Database (Oxford). 2022 Mar 9;2022:baac015. doi: 10.1093/database/baac015 (PMC9216539; doi:10.1093/database/baac015)
Supplement: baac015_Supp [file baac015_supp.zip › Supplementary File 1.docx]

**Supplementary File 1**

**An overview of current web-based resources on fibrosis research**

In order to gain a more comprehensive view of fibrosis investigations, we systematically searched for fibrosis-related resources (databases, platforms or tools) on PubMed and Google. A total number of 24 web-based resources were found published or established from 2002 to 2021. A feature comparison among these resources is illustrated in Supplementary Table 1. According to the searching results, these resources cover a wide range of fibrosis researches, including basic experiments (CyFi-MAP, Fibromine, IPF Cell Atlas, PulmonDB, Mouse Kidney Fibromics Browser, ILDgenDB, RespiratoryGenomics, Library of Molecular Associations and LiverFibrosis), clinical researches (Cystic Fibrosis Cloud Database, Participation Program for Pulmonary Fibrosis, REGIS, Cystic Fibrosis DataBase, CFTR2 and UK CF Database), disease prediction algorithm (Chronic Hepatitis B Liver Fibrosis Calculator and Cardiac Atalas Project), genetics (Cystic Fibrosis Mutation Database, CFTR2 and The CFTR Mutations Database), pharmacology (Comparative Toxicogenomics Database and Dr AFC) and candidate gene curation (TiRe, FibroAtlas, ILDgenDB and Fibrosis-related Cytokines on PubMed). Most of them concentrated on either unique types of diseases such as cystic fibrosis and idiopathic pulmonary disease, or on specialized fields of research or techniques, including cell identification, drugs and mutations. If taken website accessibility into account, only Fibromine, IPF Cell Atlas and PulmonDB contain omics-level evidence, however, these resources are dedicated merely in the area of pulmonary diseases. Apparently, the number of public resources available to provide integrated information regarding fibrosis is very limited. Therefore, as fibrosis involves a complicated bio-pathological process, it is imperative that we integrate a wider range of fibrosis-related evidence, and investigate this condition from a more comprehensive perspective.

| **Supplementary Table 1. Feature comparison among web-based resources on fibrosis research.** | | | | | | |
| --- | --- | --- | --- | --- | --- | --- |
| **Name** | **Field** | **Content** | **Date** | **Website** | **Current State** | **Reference** |
| CyFi-MAP | cystic fibrosis | up-to-date information on CFTR-related pathways and disease mechanisms | 2021 | https://cysticfibrosismap.github.io/ | Normal | Pereira C, et al. (1) |
| Fibromine | pulmonary fibrosis | multi-omics data and mining tool for target discovery in pulmonary fibrosis | 2021 | http://www.fibromine.com/Fibromine/ | Normal | Fanidis D, et al. (2) |
| TiRe | wound healing, pulmonary fibrosis (murine model) | manually curated collection of genes related to wound healing and bleomycin-induced lung fibrosis | 2021 | http://www.tiredb.org | Normal | Toren D, et al. (3) |
| Comparative Toxicogenomics Database (CTD) | multiple diseases (including IPF and cystic fibrosis) | toxicological information for chemicals, genes, phenotypes, diseases, and exposures which affect human health | 2021 | http://ctdbase.org | Normal | Davis AP, et al. (4) |
| Dr AFC | anti-fibrosis drugs | drug repositioning prediction and information based on anti-fibrosis characteristic | 2021 | https://www.biosino.org/drafc | Normal | Wu D, et al. (5) |
| IPF Cell Atlas | IPF | web-based data visualization and exploration tool for IPF single-cell datasets | 2020 | http://www.ipfcellatlas.com/ | Normal | Neumark N, et al. (6) |
| PulmonDB | IPF, COPD | integrated transcriptomic data and curated annotations for pulmonary diseases | 2020 | http://pulmondb.liigh.unam.mx/ | Normal | Villaseñor-A AB, et al. (7) |
| FibroAtlas | fibrosis-related diseases | fibrosis-related gene information from PubMed literature mining | 2019 | http://biokb.ncpsb.org/fibroatlas/ | Inaccessible | Liu J, et al. (8) |
| Mouse Kidney Fibromics Browser | renal fibrosis (murine model) | multi-omics data of mouse renal fibrosis | 2019 | http://hbcreports.med.harvard.edu/fmm/ | Inaccessible | Pavkovic M, et al. (9) |
| ILDgenDB | ILD | disease candidate genes information and associations with miRNA and SNPs by literature mining | 2018 | http://14.139.240.55/ildgendb/index.php | Inaccessible | Mishra S, et al. (10) |
| Cystic Fibrosis Cloud Database | cystic fibrosis | data from clinical studies and microbiological analysis obtained from the respiratory tract of cystic fibrosis patients | 2016 | http://servoy.infocomsa.com/cfc_database | Inaccessible | Prieto CI, et al. (11) |
| Chronic Hepatitis B Liver Fibrosis Calculator | hepatic fibrosis | algorithm to predict the presence or absence of liver fibrosis and cirrhosis | 2015 | https://www.chb-lfc.com | Normal | Salkic NN, et al. (12) |
| Participation Program for Pulmonary Fibrosis (P3F) | pulmonary fibrosis | clinical data and research information of pulmonary fibrosis | 2014 | http://www.pulmonaryfibrosisresearch.org/ | Normal | Belkin A, et al. (13) |
| Romanian National Registry for Interstitial Lung Diseases and Sarcoidosis (REGIS) | ILD | clinical data and research information of interstitial lung diseases | 2014 | https://www.regis.ro | Normal | Strâmbu I, et al. (14) |
| Cystic Fibrosis DataBase (CFDB) | cystic fibrosis | overview of the available evidence in clinical research in cystic fibrosis | 2014 | https://www.inetflow.it/CFDB | Normal | Buzzetti R, et al. (15) |
| RespiratoryGenomics | respiratory diseases (including pulmonary fibrosis and cystic fibrosis) | transcription factor regulations and related genomic features of disease-associated genes | 2012 | http://www.respiratorygenomics.com | Inaccessible | Chowdhary R, et al. (16) |
| Clinical and Functional Translation pf CFTR (CFTR2) | cystic fibrosis | CTFR variants associated enotype-phenotype information based on CTFR1 database | 2011 | https://www.cftr2.org/index.php | Normal | - |
| Cystic Fibrosis Mutation Database (CFTR1) | cystic fibrosis | up-to-date information about individual mutations in the CFTR gene | 2011 | http://www.genet.sickkids.on.ca/cftr/app | Normal | - |
| **Supplementary Table 1. (continued)** | | | | | | |
| Cardiac Atlas Project (CAP) | cardiac diseases (including cardiac fibrosis) | imaging data for computational functional analysis of cardiac diseases | 2011 | https://www.cardiacatlas.org | Normal | Fonseca CG, et al. (17) |
| The CFTR Mutations Database (UMD-CFTR) | cystic fibrosis | annotation and analysis of CTFR mutations, variations, haplotypes, complex alleles, genotypes and phenotypes | 2010 | http://www.umd.be/CFTR/ | Normal | Bareil C, et al. (18) |
| Library of Molecular Associations | hepatic diseases (including hepatic fibrosis) | validated molecular associations for hepatic diseases obtained from both PubMed literatures and microarray datasets | 2010 | https://www.medicalgenomics.org/databases/loma/news | Inaccessible | Buchkremer S, et al. (19) |
| LiverFibrosis | hepatic fibrosis (murine model) | transcriptomic data of hepatic fibrosis | 2006 | http://LiverFibrosis.nchc.org.tw:8080/LF | Inaccessible | Su LJ, et al. (20) |
| Fibrosis-related Cytokines on PubMed (FCP) | fibrosis-related diseases | fibrosis-related cytokines curated from PubMed literatures | 2003 | http://fibro.biobitfield.com/fcp.php | Inaccessible | Atamas SP, et al. (21) |
| UK CF Database | cystic fibrosis | clinical information of cystic fibrosis in the UK | 2002 | http://www.cystic-fibrosis.org.uk | Inaccessible | McCormick J, et al. (22) |
| Abbreviations: IPF - idiopathic pulmonary fibrosis; ILD - interstitial lung disease; COPD - chronic obstructive pulmonary disease. | | | | | | |

**Supplementary File References**

1. Pereira C, Mazein A, Farinha CM, et al. CyFi-MAP: an interactive pathway-based resource for cystic fibrosis. Sci Rep. 2021;11(1):22223.
2. Fanidis D, Moulos P, Aidinis V. Fibromine is a multi-omics database and mining tool for target discovery in pulmonary fibrosis. Sci Rep. 2021;11(1):21712.
3. Toren D, Yanai H, Abu Taha R, et al. Systems biology analysis of lung fibrosis-related genes in the bleomycin mouse model. Sci Rep. 2021;11(1):19269.
4. Davis AP, Grondin CJ, Johnson RJ, et al. Comparative Toxicogenomics Database (CTD): update 2021. Nucleic Acids Res. 2021;49(D1):D1138-D1143.
5. Wu D, Gao W, Li X, et al. Dr AFC: drug repositioning through anti-fibrosis characteristic. Brief Bioinform. 2021;22(3):bbaa115.
6. Neumark N, Cosme C Jr, Rose KA, et al. The Idiopathic Pulmonary Fibrosis Cell Atlas. Am J Physiol Lung Cell Mol Physiol. 2020;319(6):L887-L893.
7. Villaseñor-Altamirano AB, Moretto M, Maldonado M et al. PulmonDB: a curated lung disease gene expression database. Sci Rep. 2020;10(1):514.
8. Liu J, Sun D, Liu J, et al. FibroAtlas: A Database for the Exploration of Fibrotic Diseases and Their Genes. Cardiol Res Pract. 2019;2019:4237285.
9. Pavkovic M, Pantano L, Gerlach CV, et al. Multi omics analysis of fibrotic kidneys in two mouse models. Sci Data. 2019;6(1):92.
10. Mishra S, Shah MI, Sarkar M, et al. ILDgenDB: integrated genetic knowledge resource for interstitial lung diseases (ILDs). Database (Oxford). 2018;2018:bay053.
11. Prieto CI, Palau MJ, Martina P, et al. Cystic Fibrosis Cloud database: An information system for storage and management of clinical and microbiological data of cystic fibrosis patients. Rev Argent Microbiol. 2016;48(1):27-37.
12. Salkic NN, Cickusic E, Jovanovic P, et al. Online combination algorithm for non-invasive assessment of chronic hepatitis B related liver fibrosis and cirrhosis in resource-limited settings. Eur J Intern Med. 2015;26(8):628-34.
13. Belkin A, Fier K, Albright K, et al. Protocol for a mixed-methods study of supplemental oxygen in pulmonary fibrosis. BMC Pulm Med. 2014;14:169.
14. Strâmbu I. REGIS--Romanian National Registry for Interstitial Lung Diseases and Sarcoidosis: launch of the website and building-up the database. Pneumologia. 2014;63(2):96-9.
15. Buzzetti R, Cirilli N, Minicucci L, et al. Cystic fibrosis database (CFDB): a new web-based tool for cystic fibrosis specialists. Pediatr Pulmonol. 2014;49(9):938-40.
16. Chowdhary R, Tan SL, Pavesi G, et al. A database of annotated promoters of genes associated with common respiratory and related diseases. Am J Respir Cell Mol Biol. 2012;47(1):112-9.
17. Fonseca CG, Backhaus M, Bluemke DA, et al. The Cardiac Atlas Project--an imaging database for computational modeling and statistical atlases of the heart. Bioinformatics. 2011;27(16):2288-95.
18. Bareil C, Thèze C, Béroud C, et al. UMD-CFTR: a database dedicated to CF and CFTR-related disorders. Hum Mutat. 2010;31(9):1011-9.
19. Buchkremer S, Hendel J, Krupp M, et al. Library of molecular associations: curating the complex molecular basis of liver diseases. BMC Genomics. 2010;11:189.
20. Su LJ, Hsu SL, Yang JS, et al. Global gene expression profiling of dimethylnitrosamine-induced liver fibrosis: from pathological and biochemical data to microarray analysis. Gene Expr. 2006;13(2):107-32.
21. Atamas SP. FCP (http://fibro.biobitfield.com/fcp.php): a bioinformatic tool assisting in PubMed searches for literature on fibrosis-related cytokines. Arthritis Rheum. 2003;48(7):2083-4.
22. McCormick J, Green MW, Mehta G, et al. Demographics of the UK cystic fibrosis population: implications for neonatal screening. Eur J Hum Genet. 2002;10(10):583-90.
